# Exploring cadmium bioaccumulation and bioremediation mechanisms and related genes in soil microorganisms: a review

**DOI:** 10.1007/s11356-025-36802-9

**Published:** 2025-08-14

**Authors:** Nataly J. Galan-Freyle, Yani Aranguren-Diaz, Susana L. Ospina-Maldonado, Paula F. Chapuel-Aguillon, Maria F. Pertuz-Peña, Samuel P. Hernandez-Rivera, Leonardo C. Pacheco-Londoño

**Affiliations:** 1 Life Science Research Center, CICV, Universidad Simon Bolivar, 080002 Barranquilla, Colombia; 2 Research and Innovation in Biodiversity and Climate Change Center, Adaptia, Universidad Simon Bolivar, 080002 Barranquilla, Colombia; 3 ALERT DHS Center of Excellence for Explosives Research, Department of Chemistry, University of Puerto Rico, 00681 Mayagüez, Puerto Rico; 4 Faculty of Basic and Biomedical Sciences, Microbiology Program, Universidad Simon Bolivar, 080002 Barranquilla, Colombia

**Keywords:** Cadmium contamination, Microorganisms, Soils, Genes, Enzymes, Bioremediation, Bioaccumulation

## Abstract

Cadmium (Cd) is a highly toxic heavy metal that poses serious environmental and health risks. It reduces soil fertility and can cause renal failure, liver damage, bone fractures, hypercalciuria, and cancer in humans. Cd contamination in soil, originating from both natural and human activities, is especially concerning because it bioaccumulates in plants, entering the food chain and affecting crops like tomatoes, rice, cocoa, and lettuce. Understanding the mechanisms of Cd bioaccumulation and bioremediation in microorganisms isolated from Cd-contaminated soils is crucial for developing effective strategies to mitigate Cd contamination in soils. This review highlights recent studies on the mechanisms of Cd uptake and detoxification in microbes, emphasizing genes and enzymes that mediate Cd response. Microbial species such as *Pseudomonas aeruginosa*, *Burkholderia sp.*, and *Bacillus subtilis*, along with various fungal species, show resistance mechanisms influenced by genes that enhance their Cd tolerance. Enzymes like peroxidases, ATPase, and sucrose play roles in Cd stress responses. Key genes such as *czcA*, *czcD*, *zntA*, *cadA*, and *cadD* encode proteins that improve their tolerance to Cd. These microbial mechanisms offer sustainable solutions to improve soil health, crop productivity, and environmental safety. Future research should focus on engineering microorganisms with improved Cd-binding mechanisms, optimizing their effectiveness across different soil types, pH levels, and exposure durations.

## Introduction

Cadmium (Cd) is a heavy metal present in soil due to natural processes such as the Earth’s degassing, the weathering of parent rocks, and volcanic activities (Kierczak et al. 2021; Joya-Barrero et al. 2023). However, since the Industrial Revolution, several ecosystems have become contaminated with heavy metals, such as Cd. This heavy metal enters the soil through fertilizers, industrial wastewater, emissions, mining operations (especially Pb–Zn exploitation), coal combustion from metallurgical industries, agricultural activities, cement production, incineration, and other anthropogenic sources (Pacyna et al. 2007; Zhao et al. 2020; Bouida et al. 2022; Azhar et al. 2022).

Cd pollution in soil has become an environmental and public health problem because it is persistent and non-degradable (Khan et al. 2017; Satarug 2018; Cao et al. 2024). The main source of human exposure to Cd is ingesting food contaminated with Cd (Zou et al. 2021). Chronic Cd exposure in humans may induce renal failure, bone demineralization, increased fracture risk, liver damage, hypercalciuria, lung malfunction, decreased muscle strength, learning deficits, and neurological disorders by affecting synaptic neurotransmission; Cd accumulation might also result in cancers, such as lung, pancreas, kidney, breast, and prostate (Nordberg et al. 2018; Joshi 2021; Wu et al. 2023).

**Fig. 1:**
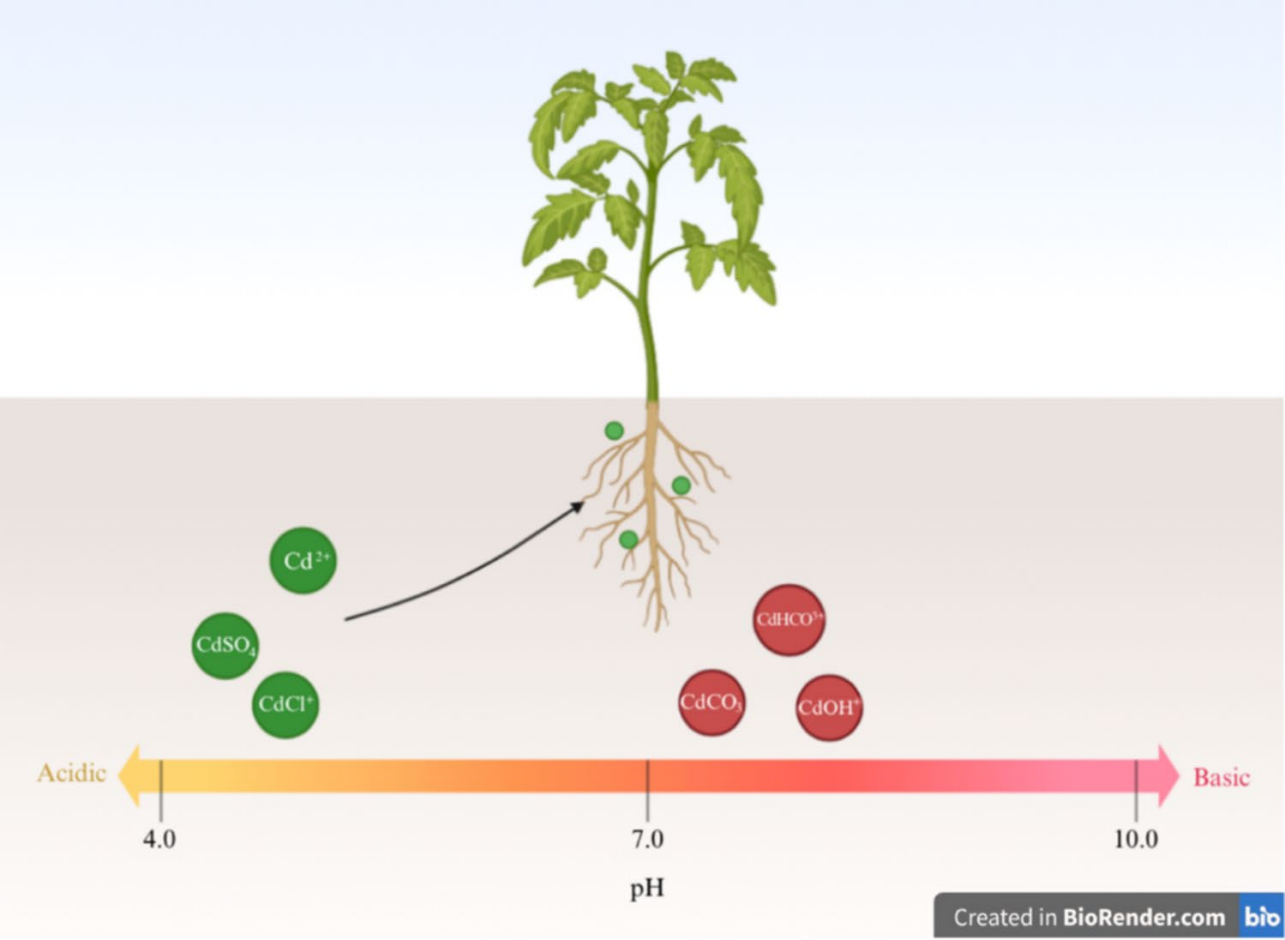
Cd bioavailability depending on soil pH range conditions. Cd is available for plants with a pH between 4.0 and 4.5. Otherwise, plants cannot absorb it when the soil pH is > 7.5

Cd transfers from soil to plants and accumulates in plant tissues, such as roots, fruits, seeds, and leaves (Baldantoni et al. 2016; Carvalho et al. 2018), becoming part of the food chain. Cd is taken up by plants and transported via membrane transporters for nutrients such as Fe^2+^, Mn^2+^, and Zn^2+^ due to similarities in their physicochemical properties. These transporters might be NRAMP1 or OsNRAMP5 for Mn^2+^, IRT1 for Fe^2+^, and OsZIP5 or OsZIP9 for Zn^2+^ (Vert et al. 2002; Cailliatte et al. 2010; Sasaki et al. 2012; Tan et al. 2020; Zhao et al. 2022). Soil pH plays a significant role in Cd bioavailability. Specifically, Cd solubility increases in acidic soils (pH 4.0–4.5), where it is present as Cd^2+^, CdCl^+^, or CdSO_4_; in contrast, it is less soluble in an alkaline pH (> 7.5), where its form is CdOH^+^, CdHCO^3+^, or CdCO_3_ (Shahid et al. 2017) (Fig. 1). Besides, plant root exudates increase Cd mobility in soils by reducing the pH, changing the redox potential, and forming complexes with organic ligands. In this process, lime could reduce Cd bioavailability, resulting in the formation of a complex set of factors that influence the availability of trace elements (He et al. 2021). These factors include elemental concentrations, pH, pCO_2_, pO_2_, redox potential, organic ligand concentrations, and microorganisms. Dissolved organic matter that comprises hydrophilic components (e.g., organic acids, carbohydrates, amino acids, and amino sugars) and hydrophobic components (aromatic phenols, hydrocarbons, fats, and nucleic acids) is one of the key factors influencing heavy metal mobility and phytotoxicity in soils (Kaiser et al. 2002). These organic compounds can form soluble organometallic complexes or be adsorbed to the soil surface rather than metals, reducing metal adsorption to the soil surface and favoring their availability to plants. In addition, Cd accumulation in soils depends on soil and rock type, geologic age, and geomorphic type (Zhao et al. 2020).

The presence of Cd affects soil fertility, leading to plant stress (reactive oxygen species (ROS) generation), and can disrupt morphology (lower biomass), alter nutrient uptake, membrane permeability, chlorosis, and inhibit plant growth (Smeets et al. 2008; Hossain et al. 2010; Bouida et al. 2022). In contrast, some algae and plants accumulate Cd and can be used as indicators of Cd-contaminated soils or phytoremediation agents, such as *Malva rotundifolia*, *Linum usitatissimum*, *Populus* spp., and *Helianthus annuus* (Wu et al. 2018; Niu et al. 2023). However, this accumulation capacity of plants also becomes a problem for human health, particularly in crops such as rice, mung beans, wheat, lettuce, cocoa, and potatoes, due to the potential for human consumption (Hossain et al. 2010; Rizwan et al. 2016; Satarug 2018; Lavres et al. 2019).

Different physicochemical methods are commonly used to mitigate Cd pollution in soils, including chemical precipitation, ion exchange, and electrochemical treatment (Dermont et al. 2008). However, these techniques produce secondary pollutants that affect soil quality (Kumar et al. 2021). In contrast, organic amendments made from sugarcane press mud, rice husk biochar, and cotton stick biochar facilitate Cd precipitation. These methods can bind toxic metals, decreasing Cd bioavailability to plants; meanwhile, they improve the soil properties by realizing nutrients (Khan et al. 2017; Rehman et al. 2020). Although organic methods are commonly used to enhance soil microbial activity and improve chemical retention capacity (Ding et al. 2023), they can contain chemicals used in agriculture or alter the concentration of heavy metals in soil, depending on the source and amount of the organic amendments (Paradelo et al. 2024). For example, animal manure may contain certain metals, which can lead to heavy metal pollution over time (Zhao et al. 2006; Zhen et al. 2020).

Some rhizosphere microorganisms are used for the bioremediation processes of contaminated soils due to their capability to tolerate high Cd concentrations and reduce Cd bioavailability by releasing chelating agents, solubilizing metal phosphates, causing redox changes, etc. (Ji et al. 2012; Li et al. 2019; Xia et al. 2021; Sharma et al. 2024). Some studies have shown that fungi belonging to the phylum Basidiomycota and Ascomycota are more resistant to high concentrations of Cd than bacteria, indicating that fungi play an important role in crop health under Cd stress (Guo et al. 2022; Zhao et al. 2023). Also, other investigations suggest that changes in bacterial and fungal diversity and composition due to Cd pollution in soil, may affect nutrient biogeochemical cycles, because these organisms may use their energy to maintain essential cellular processes rather than secrete extracellular enzymes to participate in remineralization (Chekidhenkuzhiyil et al. 2024). For this reason, indigenous microbial communities (consortia) that are resistant to high concentrations of heavy metals are used as an eco-friendly solution to reduce heavy metal pollution in soil (Hassan et al. 2020; Pal et al. 2020).

Microorganisms have shown great potential for Cd adsorption or removal from the soil, making them a low-cost and safer alternative than physicochemical methods. Unlike phytoremediation, which depends on growth cycles, that can take from months to years depending on the species, microbial soil remediation is faster but requires microorganisms with high tolerance and strong adaptability to different environments (Ma et al. 2023). This review aimed to identify specific mechanisms involved in Cd bioremediation and bioaccumulation in soil and seek to ascertain which enzymes and genes are responsible for these processes. The findings of this study will provide insight into the potential strategies that could be employed to address Cd soil contamination.

## Soil types and Cd contamination context

Numerous studies have shown that soil organic matter regulates the availability of cadmium (Cd). Sorption isotherm models have demonstrated that Cd adsorption capacity increases significantly with pH, particularly in the range of 4.0 to 7.7, where each unit increase in pH results in approximately a threefold increase in sorption capacity. At lower pH levels (4.0–5.0), soils generally exhibit similar capacities to adsorb Cd; however, sandy loam displays a marginally greater sorption capacity than loamy sand at higher pH values (6.0–7.0), likely due to differences in texture and surface area (Hong et al. 2023).

It has been studied that irrigation can impact soil properties in sandy loam and alter Cd (II) uptake. To test this mechanism, batch and desorption equilibrium experiments must be conducted to compare the interaction of wastewater-irrigated soils and the factors that create the retention capacity of Cd mobility and bioavailability.

Despite these insights, these interactions may lead to a nonlinear relationship between pH and Cd’s effective state in culture substrates, complicating predictions based solely on pH or organic matter content (Li et al. 2023a).

### Artificially contaminated soils

In artificially contaminated soils, an increase in pH is generally associated with an increase in negative surface charges on soil colloids (clay minerals, hydrated oxides, and organic matter), enhancing Cd adsorption and thereby, reducing its bioavailability. However, the effect of pH is not always linear. Some studies suggest that, depending on competing ions, complexation, or changes in organic matter solubility, the reduction in Cd bioavailability may plateau or even reverse beyond certain pH thresholds (Palansooriya et al. 2020).

Agricultural practices create synergistic interactions with organic matter in the soil. Research shows that these interactions are positively correlated with the total concentration of Pb and Cd. As the content of organic matter increases, the water-soluble and ion-exchange states of Pb and Cd decrease. Increasing levels of organic fertilizer may reduce the migration of Pb and Cd, improving soil management safety (Fang et al. 2023).

In this sense, the assessment of heavy metal immobilization in soils can be analyzed by local amendments, such as phosphate rock (PR), limestone (LS), and Portland cement (Cem). In Abdel-Kader et al. (2013), the amendments were mixed with the soil, and a control treatment was used for each soil with no amendment addition. As a result, it was found that the hydrating cement product enhances heavy metal precipitation on the surfaces of their particles due to soil extraction using DTPA, as a chelating agent, which increases the incubation period and pH.

In contrast with sandy soils, clay soils have been reported to have a high absorption capacity for heavy metals, such as Cd and Pb. This is due to the geochemical affinity of certain elements that react with soil constituents, such as structures like inner spheres. However, Cd tends to be less and more weakly adsorbed by different soils, which facilitates its leaching (Wang And Zhang 2021).

### Naturally contaminated soils

In naturally contaminated or long-term Cd-affected soils, plant-soil interactions introduce further complexity. Root exudates, plant uptake, and the dilution effect (biomass dilution of Cd concentrations) all impact the effective state of Cd (Haider et al. 2021).

Studies on lettuce roots have shown that there was an antagonistic relationship between Zn and Cd absorption in plants. The presence of other nutrient minerals with similar chemical properties might influence this synergy between the absorption mechanisms in plants (Hou et al. 2021). For example, in calcareous soils, the available content of Cd has been observed to decrease with increasing pH over a broad range (3.40–8.97), indicating a significant negative correlation. Factors affecting organic matter include Eh, CEC, anions/cations, and the state of Cd in the culture substrate; this suggests site-specific or matrix-specific behaviors (Gu et al. 2022).

### Amendments soil type and microbial Cd interaction

Microbial communities, especially arbuscular mycorrhizal fungi (AMF), play a key role in modulating Cd bioavailability. The inoculation of AMF with compost significantly reduced Cd accumulation in cacao stems and improved plant growth in contaminated soils. These effects likely result from altered rhizosphere chemistry enhancing nutrient uptake. However, microbial responses are often nonlinear and site-specific, depending on soil redox conditions, pH, and organic matter (Vallejos-Torres et al. 2022).

Certain microbial taxa have been linked to soil multifunctionality, including *Aggregicoccus* (bacteria), Sordariomycetes (fungi), *Glomus* (arbuscular mycorrhizal fungi), and *Bursaphelenchus* (nematodes). These organisms are strongly associated with soil health and resilience (Gough et al. 2020). This interaction among microbial communities is particularly relevant, as it fosters symbiotic mutualisms that enhance the cycling and availability of essential soil nutrients such as nitrogen (N) and phosphorus (P), thereby helping to alleviate nutrient deficiencies commonly found in Cd-contaminated soils.

For instance, in vitro co-culture assays involving bacterial suspensions and root pathogen systems, such as soybean and *Fusarium solani*, demonstrated that the presence of beneficial bacteria suppresses pathogen activity by inhibiting mycelial growth and spore germination. Moreover, soils amended with carboxymethyl cellulose (CMC) supported a bacterial community that exhibited enhanced resistance to cadmium (Cd) stress, suggesting that such amendments may improve microbial tolerance and overall soil function under heavy metal contamination (Cheng et al. 2023).

## Microorganisms associated with Cd bioremediation and bioaccumulation in soils

The continuous search for potential microorganism strains that allow bioremediation and bioaccumulation as an alternative solution against environmental contamination is promising, considering that only a small proportion of the representative diversity of soil microorganisms has been thoroughly studied. Due to their high adaptability, microbial populations can encounter and survive in heavy metal-contaminated environments (Abbas et al. 2018). Specific microorganisms can grow in environments with elevated metal concentrations, resulting in intrinsic tolerance mechanisms or tolerance induction by other factors in the medium, such as pH or redox potential.

Cordoba-Novoa et al. (2021) identified *Burkholderia* sp. NB10 and *Pseudomonas aeruginosa* NB2, which were isolated from contaminated cocoa soils in Colombia, demonstrate the potential for Cd reduction and resistance. These species were highly efficient at resisting 18 mg/kg of Cd, affecting its growth but recovering in the stationary phase, demonstrating that they generate an average Cd reduction of > 1 unit in the logarithmic phase. Compared to the control without Cd, the Minimum inhibitory Concentration (MIC) was 140 mg/kg, showing their potential to be used in bioremediation processes of agricultural soils contaminated with heavy metals.

Yan et al. (2024) demonstrated that the strains *Burkholderia* sp. 1–22 and *Bacillus* 6–6, which were isolated from rice rhizosphere soil and are highly tolerant to Cd, significantly enhanced the soil dry weight and effectively mitigated the negative impact of Cd on seedling growth. Although both strains showed biomass reduction when grown in high Cd concentrations, strain 1–22 exhibited greater tolerance to Cd than strain 6–6. The tolerance to high Cd levels could be investigated using soil microorganisms with the potential to form symbiotic relationships. One such microorganism is *Cupriavidus necator*, which was isolated from the rhizosphere of the legume *Sesbania virgata*. This strain demonstrates efficiency in symbiosis and immense potential to revegetate degraded areas, and the ability to bioaccumulate Cd ions as the metal concentrations increase without causing the loss of living cells in highly contaminated soils (Ferreira et al. 2018).

Similarly, microorganisms can undergo morphological changes when interacting with Cd. Jaafar et al. (2016) indicated that *Shewanella oneidensis*, isolated from soil collected from the Al-Zubair District, Basra Governorate, Southern Iraq, accumulates Cd and causes morphological changes to themselves. Depressions, shrinkage, and distortions were generated in the cell walls, as observed by transmission electron microscopy (TEM). Similarly, biosorption experiments demonstrated that *S. oneidensis* exhibits a notable capacity for Cd accumulation. This capacity is influenced by the metal ion concentration and the contact time. The results indicate a high level of bioaccumulation, particularly at metal concentrations between 50 and 100 mg/L.

Zhang et al. (2019) studied *Burkholderia cepacia* GYP1 and emphasized the importance of the mechanisms of Cd bioaccumulation under oligotrophic conditions. This strain was isolated from soil contaminated with heavy metals. According to the results obtained in this study, Cd accumulation in the outer membrane started on the first day, and intracellular Cd increased and was stable after 2 days. The largest amount of Cd was located extracellularly, reaching an impressive bioaccumulation capacity of 116 mg Cd/g dry weight.

### Experimental and operational conditions for assessing intracellular Cd levels

The mechanisms associated with Cd bioremediation can be studied through certain operational and experimental conditions that exhibit the intracellular concentration, which influences Cd-ion binding (Jia et al. 2021). Variables such as range of Cd concentration, pH, temperature, incubation time, presence of siderophores as a chelating agent, the source of the strain isolation, and the cell machinery play key roles in determining metal-binding affinity.

In another study was identified *Bacillus subtilis* as a well-characterized bacterium with notable adaptability to heavy metal stress. Under high Cd concentrations, the strain demonstrated increased production and secretion of siderophores, which significantly decreased Cd bioavailability through enhanced adsorption efficiency due to their strong binding affinity (Khan et al. 2020). This experimental framework aimed to determine the optimal conditions for evaluating intracellular Cd accumulation and the chelating role of *Bacillus subtilis* siderophores. The study systematically examined the effects of Cd concentration, pH, temperature, and incubation time. *B. subtilis* was cultured in media containing Cd^2+^ concentrations ranging from 40 to 90 μM (4.5–10.12 mg/L), incubated at 30 °C and 150 rpm for 24 h. To assess pH influence, LB broth was adjusted to values between 4.0 and 7.0, maintaining a constant Cd^2+^ concentration of 50 μM. Incubation time trials were conducted at 24, 28, 32, 36, and 48 h under consistent conditions. These controlled setups enabled a comprehensive evaluation of Cd adsorption dynamics and siderophore-mediated chelation. Notably, the results demonstrated that siderophore supplementation significantly reduced intracellular Cd levels, establishing its effectiveness as a metal-chelating agent.

The adsorption behavior of the symbiotic system and the molecular regulation mechanism of extracellular proteins in the adsorption of heavy metals have not been reported in detail.

Li et al. (2023a, b) isolated seven strains of hydroxamate siderophore-producing, Cd-tolerant endophytic bacteria from the roots of selected dominant woody plants in Cd-contaminated areas. Among them, were *Burkholderia* spp. and *Herbaspirillum* spp. These bacteria can promote plant growth under Cd stress, improving plant health and their ability to absorb Cd from contaminated soils. They also produce siderophores, which increase Cd bioavailability and aid in the metal uptake by plants. These genera are efficient in phytoremediation, contributing to the cleanup of contaminated soils by accumulating Cd in plant tissues. Experiments were carried out in pots, demonstrating that the strains had growth-promoting effects. Soil inoculation with the strains increased the acid-soluble Cd concentrations and reduced the other insoluble Cd concentrations in soils with various contamination levels, indicating that the strains could activate soil Cd. Therefore, these strains are expected to be applied as remediation agents for the endophyte-assisted phytoremediation of Cd-contaminated soils. The use of these microorganisms will depend on several factors and have significant differences in Cd reduction, absorption, tolerance, and impact on soil and plant growth, MIC concentration, and resistance mechanisms, which influence the bioaccumulation and bioremediation mechanisms (see Table 1).

**Table 1:** Microorganisms associated with Cd bioremediation and bioaccumulation in soils and their Cd tolerance, impact on soil/plant tolerance, resistance mechanisms, and MIC concentration

Bacteria	Source/ isolation	Impact on soil/plant tolerance	Resistance mechanisms	MIC concentration	References
*P. aeruginosa* NB2	Cacao-cultivated soils with Cd	Reduce stress in plants caused by the presence of Cd in soils	Extracellular Cd outflow mechanisms	140 mg/Kg	Cordoba-Novoa et al. (2021)
*Bacillus* sp. 6–6 and *Burkholderia* sp. 1–22	Rice rhizosphere soil	Promote the conversion of free Cd to non-absorbable forms. They increase the activity of antioxidant enzymes in rice, increasing the aboveground dry weight	Possess functional groups (-OH, C-O, C = O, and -NH) that participate in Cd adsorption	200 mg/L	Yan et al. (2024)
*C. necator*	Legume (*Sesbania virgata*)	Zn and Cd bioaccumulation from contaminated soils	Increase in pH to reduce toxic effects of heavy metals and production of granular deposits in their cytoplasm to accumulate Cd	9.140 μmol Zn + Cd	Ferreira et al. (2018)
*Burkholderia contaminans*	White oak (*Quercus glauca*)	Dissolve P, produce IAA, nitrogenase, and ACC deaminase promoting the growth of slash pine (*Pinus elliottii*) under Cd stress	Resistant genes, such as *czcD*, export Cd from the cytoplasm/periplasm	9.0 mM	Li et al. (2023a, b)
*Herbaspirillum* huttiense subsp. Putei	Indian azalea (*Rhododendron simsii*)		Accumulation of metal ions in the periplasm and binding of Cd onto the cell envelope through lipopolysaccharide and lipoprotein	3.0 mM	

## Mechanisms of Cd bioremediation

### Passive mechanisms

**Fig. 2:**
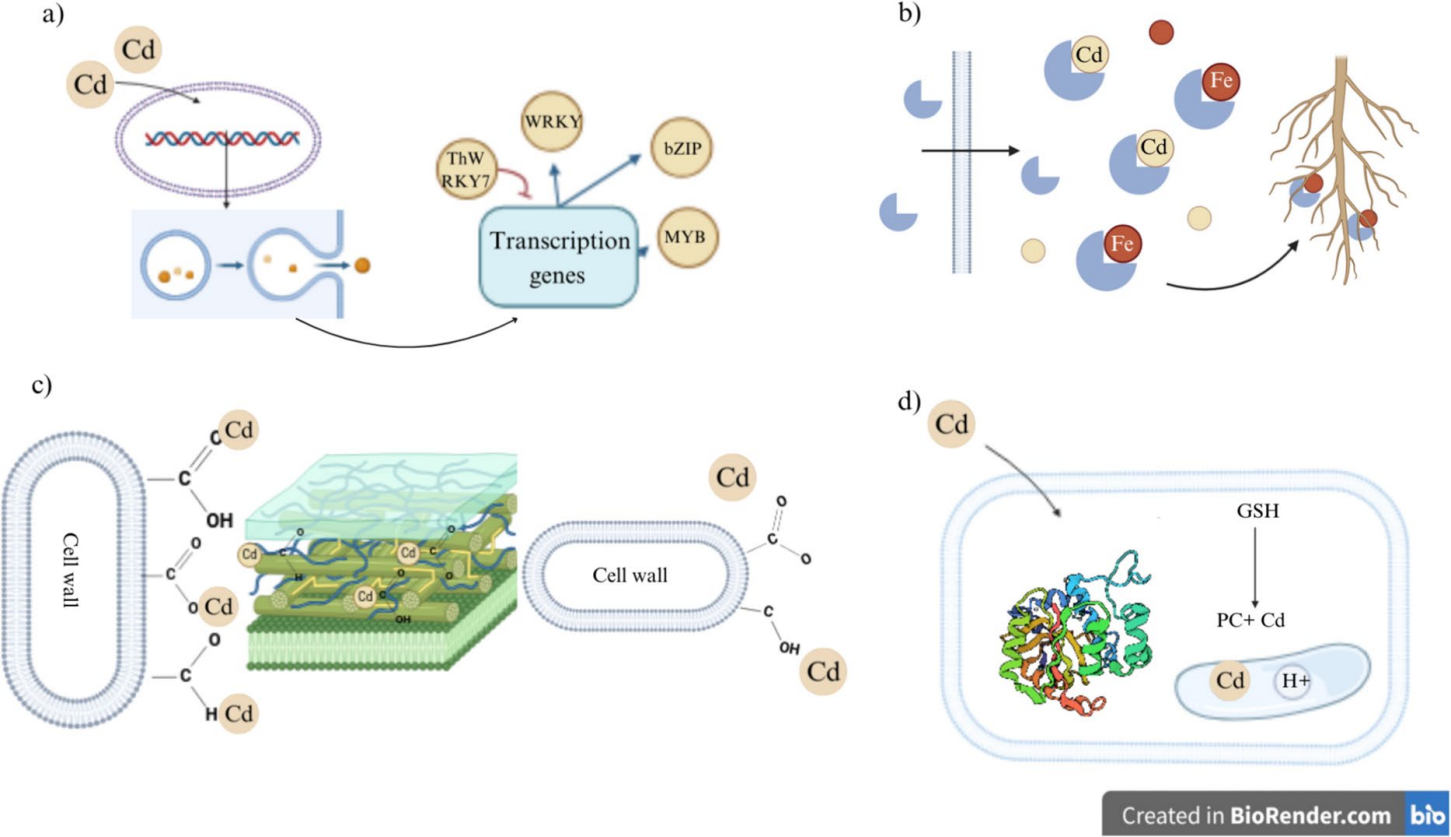
Passive mechanisms of bioremediation of Cd-polluted soils.** (a)** Extracellular mechanism. **(b)** Siderophores. **(c)** Polysaccharide functional groups. **(d)** Destruction of complex Cd-L-g-glutamyl-L-cysteinyl-glycine protein PDB: 2BTW

Passive mechanisms are represented by extracellular polymeric substances (EPS) secreted by microorganisms. This complex mixture of biopolymers consists mainly of proteins, polysaccharides, and nucleic acids, can bind various metalloids through coordination and complexation (Gu et al. 2023). In this sense, extracellular Cd from the cell wall enters the DNA machinery and processes, inducing vesicle production, which exhibits gene transcription factors that are gene-stress responses to the presence of Cd (Fig. 2a). The addition of EPS can impact the physical and chemical properties of soil. An example of this was reported by Lian et al. (2022), in this case, the stress induced by Cd(NO_3_)_2_ improved the content of EPS from *Pseudomonas aeruginosa* and *Alcaligenes faecalis*, and the adsorption capacity of Cd (II) reached 232.45 and 300.20 mg/g EPS, respectively.

Another significant passive mechanism are siderophores that can chelate metal ions. These organic compounds have a strong affinity for Fe but can bind to other metals such as Cd, Pb, Al, As, Mn, Ni, Zn, and Co (Gaonkar And Bhosle 2013). Siderophores can bind to Cd and prevent it from entering the cell (Syed et al. 2023). Yadav et al. (2023) evaluated the capacity of *Trichoderma harzianum* siderophore to reduce Cd stress. The results indicated that the siderophore enhances the level of enzymatic antioxidants, including catalases, peroxidases, and superoxide dismutase, since these enzymes depend on iron as a component, promoting the growth of *Solanum melongena*; in this sense, siderophores increased Fe transportation inside the plant while immobilizing Cd, decreasing its uptake in roots (Fig. 2b).

Besides, it is fundamental to understand the mechanisms of Cd binding within polysaccharide functional groups because the root cell wall is mostly composed of polysaccharides, including hemicellulose, pectin, and cellulose, which are critical components that bind metal ions. These interact within the cell wall and can be adsorbed by surface complexation and electrostatic attraction (Ahmad et al. 2020). Meanwhile, ion transfer or exchange is the main Cd adsorption force, particularly for pectin and hemicellulose, which consist of carboxyl groups, hydroxyl groups, and amidogen (Yu et al. 2021). Most Cd^2+^ is immobilized in the hemicellulose, which has a positive correlation with Cd fixation ability, and is exhibited on the surface of COO- and COOH groups (Yu et al. 2021; Gu et al. 2023) (Fig. 2c). Furthermore, NADPH oxidase concomitant ROS generation is another proposed mechanism for Cd-induced oxidative damage (Gill And Tuteja 2010). Cd promotes the activity of plasmalemma associated with NADPH oxidase which results in catalyzing O2 reduction reaction by making use of NADPH as a reducing agent; eventually, the formation of superoxide (O2 −) free radical (Fujii et al. 2022).

Furthermore, organic complexation is a promising application in the bioremediation field due to its scalability and bioavailability, which consists of making organic complexes between Cd and organic ligands that could be organophosphorus or non-organophosphorus. Cd chelation or ion binding occurs due to compartmentalization in the vacuoles (Most And Papenbrock 2015). The interaction is possible due to the phytochelatins that are well-known as a class of heavy metal-binding peptides found in several dicotyledonous and monocotyledonous plants (Grill et al. 1987). These are synthesized from glutathione (GSH) by the enzyme phytochelatin synthase. Exposure to Cd stimulates the synthesis of phytochelatins, which rapidly form a low-molecular-weight bound with higher polymers. These complexes acquire acid-labile sulfur (S^2−^) and form a high molecular complex (Fig. 2d).

### Active mechanisms

**Fig. 3:**
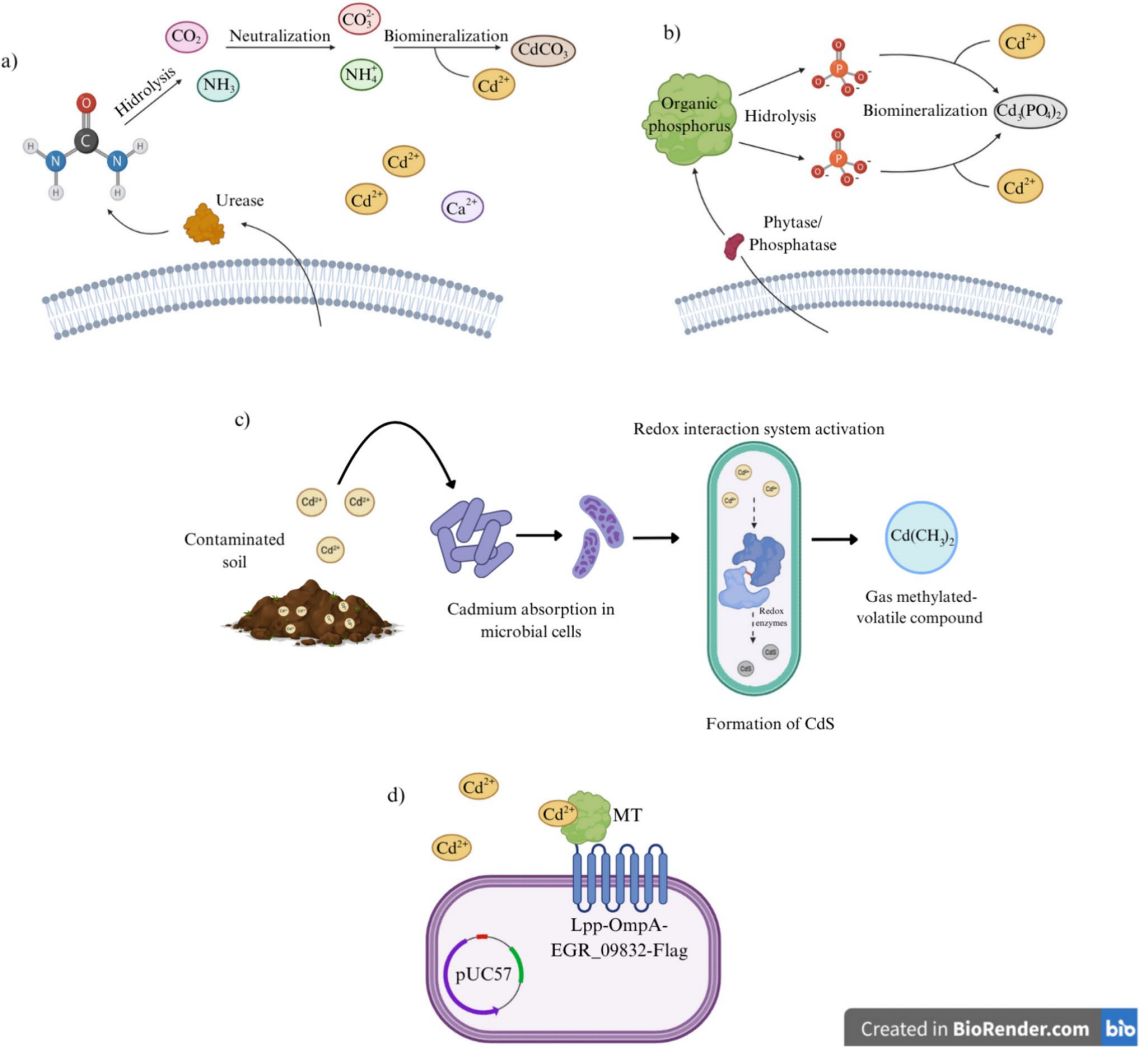
Active mechanisms of bioremediation of Cd-polluted soils.** (a)** Biomineralization-based MICP. **(b)** Biomineralization-based MIPP. **(c)** Mechanism of oxidation–reduction and volatilization of microorganisms. **(d)** MT production

Biomineralization or precipitation, metallothionein (MT) production, volatilization, and oxidation/reduction have been studied as active mechanisms in bioremediation processes. In this sense, biomineralization-based microbially induced calcite precipitation (MICP) is a process in which organisms such as bacteria play an important role in the precipitation of mineral materials. For example, ureolytic bacteria species can induce calcite precipitation for heavy metal removal. Calcite absorbs Cd (II) ions into its crystal structure, resulting in the deposition of mineral particles (Achal et al. 2012; Khadim et al. 2019) (Fig. 3a). Urease-producing microorganisms such as *Bacillus amyloliquefaciens* produce this enzyme due to its metabolic activity, leading to the precipitation of calcium carbonate (Khadim et al. 2019). Other examples include *Serratia* sp. (Diez-Marulanda and Brandão 2024), *Enterobacter* sp. (Peng et al. 2020), and *Lactobacillus sphaericus* (Kang et al. 2014). In this process, the release of CO_2_ and NH_3_ caused by urea hydrolysis increases the pH to ≥ 7.0 (Rajasekar et al. 2021). Under alkaline conditions, CO_2_ is converted into CO_3_^2−^, which binds to Ca^2+^ to form CaCO_3_ (Zhang et al. 2023). In this bioremediation method, Cd^2+^ replaces Ca^2+^ due to its ionic radius similarities, creating CdCO_3_ (Zheng et al. 2021). Another biomineralization method is microbial-induced phosphate precipitation (MIPP) (Fig. 3b). Under normal conditions, some microorganisms produce phytase or phosphatase to increase phosphate bioavailability in soils to promote plant growth (Katsalirou et al. 2016); in this case, these enzymes can be used to transform organic P substrates into inorganic anions (PO_4_^3+^) and create biomineralized phosphate with Cd ions [Cd_3_(PO_4_)_2_] (Zheng et al. 2021). Some microorganisms used to remove Cd from contaminated soils through the MIPP method include *B. subtilis* and *Sporosarcina pasteurii* (Qian et al. 2018). The capacity to produce compounds to trap heavy metals provides a bioremediation method for Cd-contaminated soils.

Various microorganisms employ mechanisms such as volatilization and redox reactions to detoxify Cd. These organisms can reduce Cd toxicity by converting it into less harmful forms or immobilizing it inside cells. The oxidation/reduction (redox) mechanism involves the transfer of electrons from one element to another (Fig. 3c). Some microorganisms can catalyze the bioremediation of heavy metals, which involves the chemical transformation of contaminants in the soil. Particularly, sulfate- and iron-reducing bacteria, such as *Geobacteraceae*, alter the oxidation state of Cd, which reduces its solubility and mobility in contaminated soils (He et al. 2019) and can also be stimulated to produce a chemically reactive redox barrier (Ramasamy et al. 2007). Likewise, Fenton-type processes involving iron oxide interactions have great potential to remove these contaminants (Garzón-Cucaita And Carriazo 2022). Metals such as Pb and Cd can be precipitated in their sulfide forms using biogenic H_2_S produced by sulfate-reducing microorganisms, such as bacteria and archaea (Rahman And Singh 2020). In contrast, the presence of biosolids in contaminated soils can facilitate Cd reduction, which is attributed to microbial activity that promotes the metal’s chemical transformation (Ruiz 2011). In addition to reducing Cd toxicity, these processes also improve the soil quality by increasing its nutrient retention capacity. Volatilization is a microbial process by which heavy metals are converted into volatile compounds, enabling their release into the atmosphere and reducing their concentration in the environment. This transformation typically occurs after redox reactions, during which specific microbial enzymes reduce metal ions to more volatile forms. For example, the reduction of cadmium ions (Cd^2+^) to elemental cadmium or other volatile derivatives facilitates their volatilization (Sharma et al. 2024). Thus, redox reactions precede and enable volatilization, working together to eliminate both heavy metals and organic pollutants from contaminated sites (Meng et al. 2019)**.** Some bacteria such as *Pseudomonas* spp., *Escherichia* spp., *Clostridium* spp., and *Bacillus* spp. can convert metals, such as Cd, Hg (II), Se, As, and Pb into methylated gaseous form (Ramasamy et al. 2007).

Otherwise, MT is a cysteine-rich protein that regulates gene expression and the Cd detoxification process by binding with metal ions. It also protects cells from damage by inhibiting ROS production (He et al. 2024). *Pseudomonas putida* has shown an excellent ability to resist heavy metal pollution and participate in bioremediation processes through Cd sequestration and immobilization in soil. MT proteins from these bacteria are known as pseudothioneins (Higham et al. 1986). These MTs and other proteins in this family have negatively charged residues that bind positively charged metal ions such as Cd (Tasleem et al. 2023). He et al. (2024) fused the synthetic gene Lpp-OmpA [lipoprotein (Lpp) and outer membrane protein A (OmpA)] from *Escherichia coli* with the plasmid vector pUC57. Lpp helps anchor target proteins to the outer membrane, and OmpA transports target proteins through the outer membrane (Jeiranikhameneh et al. 2017). The plasmid pUC57-Lpp-OmpA was used to immobilize MT on the surface of *E. coli* DH5α to investigate its impact on Cd^2+^ adsorption using the *EGR_09832* gene from *Echinococcus granulosus* to express MT and create a plasmid vector pBSD-Lpp-OmpA- EGR_09832-Flag (pBSD-LEF), and consequently, a recombinant *E. coli* DH5α (pBSD-LEF) which could be used for bioremediation purposes (Fig. 3d).

## Mechanisms of Cd bioaccumulation

The uptake of different microelements, such as importer complexes that create a translocation pathway across the lipid bilayer, or import system, is known as bioaccumulation, and it operates due to the energy system’s capacity to transport ions and active functional groups that work through the membrane (Collin et al. 2022). These functional groups can transport and sequester ions inside the cytoplasm. The energy-dependent efflux mechanism is activated by an increase in intracellular Cd to transport Cd ions out of the cell and shield it from extremely damaging levels of Cd. The cells would capture or absorb extracellular Cd ions by the abundant EPS secreted simultaneously (Bramhachari And Nagaraju 2017).

Diverse macromolecules in biofilm, especially proteins, carbohydrates, and nucleic acids, can bind to Cd and incorporate it into microbial cell walls and the EPS matrix (Han et al. 2020b). The process of reducing Cd bioavailability and forming biofilm cells is associated with Cd precipitation with groups such as phosphates, carbonates, or sulfides (van Hullebusch et al. 2016). Therefore, an increased percentage of bacteria forming biofilms and improved biofilm-based Cd are significant factors in decreased bioavailability and improved Cd immobilization in inoculated soils.

The bioaccumulation characteristics of Zn^2+^ and Cd^2+^ by a novel species, *Streptomyces zinciresistens* CCNWNQ0016(T), were investigated. *S. zinciresistens* accumulated Zn^2+^ and Cd^2+^ on the cell wall, followed by intracellular accumulation. The mycelium is deformed and aggregated, forming Zn and Cd precipitate on the cell surface. Electron-dense granules were detected on the cell wall and within the cytoplasm (Lin et al. 2012). Amino, carboxyl, hydroxyl, and carbonyl groups are responsible for Zn^2+^ and Cd^2+^ biosorption. Filali et al. (2000) studied bacterial isolates from wastewater, including *P. aeruginosa*, *Klebsiella pneumoniae*, *Proteus mirabilis*, and *Staphylococcus*, which were resistant to heavy metals and antibiotics. Similarly, Sharma et al. (2000) isolated a Cd-resistant *Klebsiella*, that precipitates a significant amount of CdS. The heavy metal-resistant organism could be a potential agent for the bioremediation of heavy metal pollution. Multiple tolerances occur only to toxic compounds with similar underlying toxicity mechanisms because they are affixed to their environmental niche.

In contrast, Zhang et al. (2019) showed that Cd accumulation occurred on the outer membrane within 1 day, and that intracellular Cd increased and remained stable after 2 days. After that, the increased amount of extracellular Cd is related to the secreted EPS. As a result, this would increase intracellular Cd, and the energy-dependent efflux system takes effect to transport excessive intracellular Cd ions, which would protect themselves from highly toxic forms of Cd. Meanwhile, the cells would secrete plenty of EPS to entrap or adsorb extracellular Cd ions.

A key factor is controlling sulfur-oxidizing bacteria, which are an important bioaccumulation mechanism. One particular interest is *Thiobacillus*, which can oxidize sulfur into S_2_O_3_, S_4_O_6_, and SO_4_ under aerobic conditions (Joshi et al. 2021) by increasing the absorption and accumulation of heavy metals in plants. Although sulfur plays a beneficial role in enhancing plant tolerance to heavy metals, its application can cause severe problems (Liu et al. 2022). Therefore, it is necessary to develop low-cost environmental practices and essential plant nutrients that make sulfur and other elements ideal for phytoextraction methods.

Bacterial taxa such as *Serratia* sp., *Bacillus* sp., *Ralstonia* sp., *Enterobacter* sp., *Exiguobacterium* sp., and *Lactococcus* sp. are recognized for accumulating Cd extracellularly. Contact time and Cd concentration are truly relevant to this process. Many bacterial species use efflux pumps to keep metal ions outside the cell and prevent toxicity (dos Santos 2018). These bacteria can bind to Cd and intracellular binding proteins, such as bacterial MTs, metallochaperones, cell wall components (exopolysaccharides), or at-surface factors. For example, Cd binds to the capsular surface in some bacteria such as *Arthobacter viscosus* and *Klebsiella aerogenes*; in other bacteria, Cd binds to insoluble cell-bound Cd-HPO_4_ and is stored intracellularly in mutant *Citrobacter* (dos Santos 2018).

## Genes/enzymes associated with Cd tolerance in microorganisms

Microorganisms can employ genes that code for proteins and metabolites linked to the metal intake in the surrounding soil. Enzymes play a vital role in transporting microelements, proteins, and peptides directly to uptake, and they can use ligands that sequester heavy metals after interacting with the intracellular storage system (Diep et al. 2018). Some enzymes can induce MICP (Wang et al. 2024). Aside from this, other processes involving the interchange of ligands and ions include biosorption, which involves ion exchange, physical adsorption, and bioaccumulation on microbial cell walls and complex formation (Fomina And Gadd 2014).

Cadmium (Cd) tolerance mechanisms are closely linked to oxidative stress responses, as elevated Cd concentrations can induce membrane lipid peroxidation, leading to cellular membrane damage (Mitra et al. 2018). Enzymes such as urease, sucrase, and peroxidase have been identified as predominant in mediating these stress responses (Yan et al. 2024). Additionally, genes such as *atPnx*, *metab*, *cadA*, and *cadD*—characterized in species like *Saccharomyces cerevisiae* and *Desulfovibrio desulfuricans*—encode enzymes including ATPases and glutathione S-transferases (GSTs), which contribute to the maintenance of intracellular Cd homeostasis (Song et al. 2004; Naz et al. 2005). Furthermore, genes such as *cadB* and *cadX*, identified in *Staphylococcus aureus*, *Alcaligenes eutrophus*, and *Ralstonia* sp. CH34, encode P1B-type ATPases that facilitate the active efflux of Cd^2+^ from the cytoplasm. These proteins often function alongside regulatory elements such as *cadC*, a transcriptional activator that induces gene expression in response to Cd exposure, thus forming a rapid and efficient detoxification system (Abbas et al. 2018).

The cadR gene, present in *Pseudomonas aeruginosa*, encodes a transcriptional regulator of the MerR/ArsR family, which detects the presence of Cd and activates the transcription of resistance genes. These regulators change their conformation upon binding to Cd^2+^, which allows them to facilitate the transcription of resistance operons such as cadA, thus promoting a rapid molecular response (Chellaiah 2018). In *E. coli* P4, genes such as czcA, mutS, ftsZ, clpB, ef-tu, and dhaK are involved in multiple processes: from active metal transport by ATPases (such as czcA) to cell cycle maintenance, DNA repair, and protein stability under stress. These proteins act synergistically to protect the cell against structural and genetic damage caused by Cd exposure (Khan et al. 2015) and the htpX gene, present in *E. coli* BL21(DE3), encoding a membrane glutathione oxidoreductase involved in Cd detoxification by recycling reduced glutathione (GSH), essential to combat oxidative stress. Its action allows the maintenance of intracellular redox homeostasis, which is critical in the face of Cd accumulation (Zhao et al. 2022).

An additional mechanism analysis of Cd bioaccumulation by GYP1, based on iTRAQ-based proteomics, revealed that Cd (II) could cause the upregulation of Cd response-related genes, such as GSH S-transferase (GST), type VI protein secretion systems, and Cd^2+^/Zn^2+^-exporting ATPase, which may help to maintain intracellular Cd homeostasis. The immobilization process includes the following steps: (1) Cd(II) is quickly immobilized on the cell surface in coordination with the functional groups; (2) Cd(II) is transported to the cells and accumulated in the cytoplasm; and (3) intracellular Cd(II) efflux depends on the energy and the extracellular Cd(II) that EPS adsorbs (Zhang et al. 2019).

Soil enzyme and gene activities are helpful bioindicators of soil quality, microbial activity, and environmental responses to contaminants. The time-course effect of OSP on Cd transporter gene expression is associated with the relative expression of genes. Some include *OsNramp5*, *OsNramp1*, *OsIRT1*, and *OsHMA2*, which were significantly down-regulated by OSP application under Cd exposure (Yang et al. 2023).

**Fig. 4:**
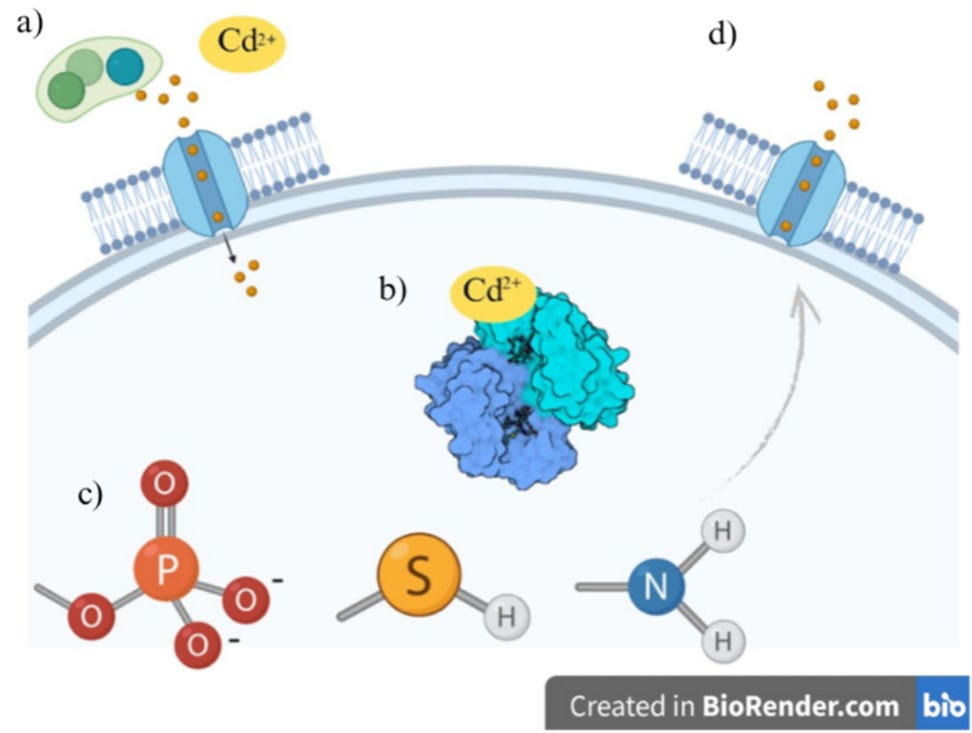
Mechanisms of Cd bioaccumulation on microbial cell walls.** (a)** Biofilm formation trapping extracellular Cd^2+^. **(b)** Cd^2+^ importer complexes on the cell membrane and intracellular sequestration by complex proteins, such as glutathione S-transferase (GST), type VI (PDB 1GNW), and the protein secretion system. **(c)** Transportation of excess intracellular Cd ions. **(d)** Exhibit: Surface functional groups such as sulfides, phosphates, and amines, on the membrane

The correlation between soil nutrient cycles, particularly those involving C, N, and P, influences different soil enzyme types, which may respond differently to biotic and abiotic stresses. This implies Cd uptake mechanisms (Bandara 2017). Sucrase plays a role in the soil’s C cycle, which is influenced by the amount of organic matter present in the soil (Schimel And Schaeffer 2012). This enzyme has a significant impact on helping regulate abiotic stress in plants when Cd is present. As a result, sucrose transporters and sugars are eventually exported by transporters that load and unload sucrose in the phloem, translocating and allocating sugar in plants. These interactions correlate with Cd uptake (Fig. 4) (Li et al. 2020).

An investigation using scanning electron microscopy-TEM, and energy-dispersive X-ray spectroscopy identified and confirmed that the Cd-resistant and plant growth-promoting *Enterobacter* sp. accumulated Cd. It improved rice seedling development and decreased Cd uptake. The findings led to the conclusion that the chosen bacterial isolates could engage in bioaccumulation activity, making them a suitable choice for bioremediation procedures. This is associated with certain enzymes, such as amylase and protease, that increase Cd availability. This is caused by the interaction between heavy metal ions that promote the coordination between the enzyme’s active site and its substrate. Similarly, superoxide dismutase is a metalloenzyme that catalyzes the dismutation of superoxide anions into H_2_O_2_, which is further detoxified into water and divalent oxygen by the enzymes, catalase and GSH peroxidase (Mitra et al. 2018).

*Bacillus* strains have demonstrated a diverse genetic repertoire associated with cadmium (Cd^2+^) resistance. Key genes such as *cadA*, *cadC*, *czcD*, and *zntA* encode P-type ATPases and heavy metal efflux transporters, which facilitate the active extrusion of Cd^2+^ from the cytoplasm. These proteins form part of conserved regulatory and transport systems that enable rapid microbial responses to heavy metal stress (Nies 2003). Moreover, oxidative stress induced by Cd^2+^ exposure activates antioxidant defense mechanisms mediated by genes such as *sodA* (superoxide dismutase) and *katA* (catalase), which mitigate cellular damage by neutralizing reactive oxygen species (Wang And Zhang 2021). Additionally, *Bacillus* species contribute to Cd^2+^ immobilization through interactions with their cell wall components, enabling mechanisms such as adsorption, bioaccumulation, and in certain instances, biomineralization. Table 2 summarizes key genes and enzymes implicated in microbial Cd resistance and their role in bioaccumulation pathways.

**Table 2:** Genes and enzymes associated with Cd bioaccumulation by microorganisms

Gene	Enzyme/protein	Mechanism	Microorganism	References
*atPrx*, *atPrx33*, *atPrx34 GSH1, GSH2, YCF1*	Urease, sucrase, peroxidase	Cd resistance	*Saccharomyces cerevisiae*	Song et al. (2004)
*metab*, *cadAC*, *cadD*	ATPase, type VI protein secretion systems, GST	Cd resistance	*Desulfovibrio desulfuricans*, *Desulfococcus multivorans*	Naz et al. (2005)
*cadA, cadC, czcD, zntA, sodA, katA*	P-type ATPase (*cadA*), transcriptional regulator (*cadC*), cation transporters (*czcD*, *zntA*)	Resist Cd stress	*Bacillus subtilis*	Moore et al. (2005)
*cadA*, *cadB*, *cadX*	ATPase	Cd resistance	*Staphylococcus aureus*, *Alcaligenes eutrophus*, *Ralstonia* sp. CH34	Abbas et al. (2018)
*cadR*	MerR/ArsR	Cd resistance	*P. aeruginosa*	Chellaiah (2018)
*czcA1A*, *czcA2A*, *czcB1A*, *czcC1A*	ATPase	Resist Cd stress	*Pseudomonas putida*	Liu et al. (2021)
*czcCBA,*	CzcA, CzcB, CzcC	Cd resistance	*Pseudomonas aeruginosa, Cupriavidus metallidurans*	Nies (2003)
*oxyR, ohrR*	Reductases, catalase, peroxide regulators (OhrR, OxyR)	Resist Cd stress	*Xanthomonas campestris*	Banjerdkij et al. (2005)
*czcA*, *mutS*, *ftsZ*, *clpB*, *ef-tu*, *dnaK*	ATPase	Cd resistance	*E. coli* P4	Khan et al. (2015)

The adsorption method is widely used due to its low cost and environmentally friendly efficiency. Microorganisms used in the biosorption process to remove Cd from the environment usually accompany the process with bioaccumulation and biomineralization (Anand et al. 2023). Han et al. (2020a, b) explored the effects of Cd on the bioaccumulation, biosorption, and photosynthesis of *Sarcodia suiae*. The study examined the ratios of phycoerythrin, phycocyanin, and allophycocyanin in response to Cd exposure. This is the response of different microorganisms that use their molecular mechanisms to combat Cd stress. In this case, Cd is an essential element for life that acts as a catalyst for enzyme metabolism. Based on this concept, Hrimpeng et al. (2006) investigated gene expression changes in *Xanthomonas campestris* in response to Cd exposure. They proved gene upregulation in the OxyR and OhrR regulon, establishing a complex regulatory pattern induced by Cd. Their research demonstrated that Cd-induced resistance to peroxides is produced by the metal’s ability to increase levels of enzymes that protect against peroxide stress, such as alkyl hydroperoxide reductase, monofunctional catalase, and organic hydroperoxide resistance protein. Cd-induced resistance to H_2_O_2_ depends on the functional OxyR, a peroxide-sensing transcription regulator.

## Future perspectives

The reviewed studies provided valuable insights into the biological mechanisms of Cd bioaccumulation and bioremediation. Understanding these processes at the molecular and genetic levels is essential for developing effective bioremediation strategies. Future research should focus on exploring the potential of genetically modified microorganisms to enhance the efficiency and sustainability of Cd removal in contaminated environments. These modifications could include genes that promote plant growth and allow Cd detection at an intracellular level.

It is important to study the enzymatic and genetic changes in microorganisms that are exposed to Cd. This will help to standardize the Cd bioaccumulation response to chemical substances and agricultural management. This review makes significant contributions to our understanding of microbial strategies for Cd remediation, particularly through the identification of *Burkholderia* sp. (Zhang et al. 2019; Cordoba-Novoa et al. 2021; Li et al. 2023b)*, Pseudomonas aeruginosa* (Ramasamy et al. 2007; Cordoba-Novoa et al. 2021; Lian et al. 2022) and *Bacillus* sp. (dos Santos 2018; Khan et al. 2020; Yan et al. 2024). These strains demonstrated notable Cd resistance and reduction capabilities, positioning them as strong candidates for bioremediation strategies. Furthermore, the application of AMF as well as other fungi species was found to be a practical method for reducing Cd bioavailability in plants and improving nutrient uptake by plants (Vallejos-Torres et al. 2022; Guo et al. 2022; Zhao et al. 2023). This not only improved plant growth under metal stress, but also indicated the critical role of microbial communities in shaping rhizosphere chemistry and nutrient dynamics. Additionally, the discovery of siderophores’ stronger binding affinity for Cd^2+^ (Khan et al. 2020) represents a mechanistic insight with significant implications for designing targeted bioremediation interventions based on metal chelation.

The investigated genes revealed the coexistence of peroxidase enzymes and genes that promote an oxidative stress response, establishing a complex regulatory pattern. In this context, future research should focus on the optimization and gene editing of microbial strains that can modulate Cd ion binding at the intracellular level. It is important to understand the effects of exposure duration and how biotechnologically modified strains can be applied not only to chronically contaminated soils but also to naturally contaminated soils and how this can cause several changes in pH, redox potential (Eh), cation exchange capacity (CEC), and the activity of various anions and cations, all of which synergistically affect Cd dynamics.

## Data Availability

The authors confirm that the data used to support the findings of this study are available as part of the article and no additional source data are required.
